# Supplementary material for: Waning of anti-SARS-CoV-2 antibodies after the first wave of the COVID-19 pandemic in 2020: A 12-month-evaluation in three population-based European studies
Source: PLoS One. 2025 May 9;20(5):e0320196. doi: 10.1371/journal.pone.0320196 (PMC12063904; doi:10.1371/journal.pone.0320196)
Supplement: S1 Table — (DOCX) [file pone.0320196.s001.docx]

**Supplementary Table 1.** **Distribution of ELISA Anti-N OD ratios according to ELISA Anti-S positivity, November 2020 *(the national EpiCov cohort, round 2).***

|  | **ELISA Anti-S IgG in November 2020** | | |
| --- | --- | --- | --- |
|  | **Negative**  **N = 141** | **Intermediate**  **N = 102** | **Positive**  **N = 356** |
| **ELISA Anti-N IgG**  **in November 2020** |  |  |  |
| **Negative (N = 351)** | 91.0 (118/130) | 80.0 (69/86) | 53.0 (164/307) |
| **Intermediate (N = 17)** | 7.7 (10/130) | 3.5 (3/86) | 1.3 (4/307) |
| **Positive (N = 155)** | 1.5 (2/130) | 16.0 (14/86) | 45.0 (139/307) |
| Missing | 11 | 16 | 49 |

% (n/N)
