# Supplementary material for: Waning of anti-SARS-CoV-2 antibodies after the first wave of the COVID-19 pandemic in 2020: A 12-month-evaluation in three population-based European studies
Source: PLoS One. 2025 May 9;20(5):e0320196. doi: 10.1371/journal.pone.0320196 (PMC12063904; doi:10.1371/journal.pone.0320196)
Supplement: S2 Table — (DOCX) [file pone.0320196.s002.docx]

**Supplementary Table 2.** **ELISA anti-N IgG OD ratios in November 2020 and Neutralizing antibodies in May 2020 *(the national EpiCov cohort).***

|  | **Anti-N IgG in November 2020** | | |  |
| --- | --- | --- | --- | --- |
| **Neutralizing antibodies in May 2020** | **Negative**  **N = 351** | **Intermediate**  **N = 17** | **Positive**  **N = 155** | **P-value** |
| **Negative** (VNT < 40) | 31.2 (105/337) | 41.2 (7/17) | 12.2 (18/147) | < 0.001 |
| **Positive** (VNT ≥ 40) | 68.8 (232/337) | 58.8 (10/17) | 87.8 (129/147) |  |
| Missing | 14 | 0 | 8 |  |

% (n/N)

Fisher’s exact test
