# Supplementary material for: Waning of anti-SARS-CoV-2 antibodies after the first wave of the COVID-19 pandemic in 2020: A 12-month-evaluation in three population-based European studies
Source: PLoS One. 2025 May 9;20(5):e0320196. doi: 10.1371/journal.pone.0320196 (PMC12063904; doi:10.1371/journal.pone.0320196)
Supplement: S3 — (DOCX) [file pone.0320196.s003.docx]

**S3. Note : Acknowledgements**

**ORCHESTRA WP3 “Population-based cohorts” working group**, Lead author: Laurence Meyer

Federica D'ambrosio, Federico Banchelli, Letizia Bartolini, Laura Bonvicini, Dmitry Bulaev, Pasquale Denardo, Costanza Dichiara, Joëlle Fritz, Pamela Matias Garcia, Paolo Giorgirossi, Michael Hölscher,

Marie-Theres Huemer, Alexey Kolodkin, Rejko Krüger, Elisabetta Larosa, Ana-Lucia Mayen-Chacon, Laurence Meyer, Aayah Nounu, Jochen Ohnmacht, Marc O'sullivan, Christina Reinkemeyer, Eduardo Rosales Jubal, Giulio Vecchia, Melanie Waldenberger

**CON-VINCE consortium**

Tamir Abdelrahman, Geeta Acharya, Gloria Aguayo, Pinar Alper, Wim Ammerlaan, François Anciens, Ariane Assele-Kama, Christelle Bahlawane, Katy Beaumont, Nadia Beaupain, Lucrèce Beckers, Camille Bellora, Fay Betsou, Luc Biver, Sandie Boly, Dirk Brenner, Henry-Michel Cauchie, Eleftheria Charalambous, Emilie Charpentier, Estelle Coibion, Sylvie Coito, Delphine Collart, Manuel Counson, Brian De Witt, Antonelle Di Pasquale, Olivia Domingues, Claire Dording, Jean-Luc Dourson, Bianca Dragomir, Tessy Fautsch, Jean-Yves Ferrand, Thibault Ferrandon, Ana Festas Lopes, Guillaume Fournier, Joëlle Véronique Fritz, Manon Gantenbein, Piotr Gawron, Laura Georges, Soumyabrata Ghosh, Stéphane Gidenne, Enrico Glaab, Clarissa Gomes, Borja Gomez Ramos, Vyron Gorgogietas, Jérôme Graas, Valentin Groues, Wei Gu, Gael Hamot, Anne-Marie Hanff, Maxime Hansen, Linda Hansen, Lisa Hefele, Laurent Heirendt, Ahmed Hemedan, Estelle Henry, Margaux Henry, Eve Herkenne, Sascha Herzinger, Christiane Hilger, Laetitia Huiart, Alexander Hundt, Judith Hübschen, Gilles Iserentant, Philipp Jägi, Anne Kaysen, Piyapong Khurmin, Fédéric Klein, Tommy Klein, Stéphanie Kler, Alexey Kolodkin, Rejko Krüger, Pauline Lambert, Jacek Jaroslaw Lebioda, Sabine Lehmann, Marie Leick, Anja Leist, Morgane Lemaire, Andrew Lumley, Annika Lutz, João Manuel Loureiro, Monica Marchese, Tainà Marques, François Massart, Patrick May, Maura Minelli, Alessandra Mousel, Maeva Munsch, Sophie Mériaux, Friedrich Mühlschlegel, Mareike Neumann, Trang Nguyen, Beatrice Nicolai, Marc Paul O'Sullivan, Leslie Ogorzaly, Jochen Ohnmacht, Christiane Olesky, Markus Ollert, Claire Pauly, Laure Pauly, Lukas Pavelka, Christian Penny, Magali Perquin, Achilleas Pexaras, Palma di Pinto, Marie France Pirard, Jean-Marc Plesseria, Guilherme Ramos Meyers, Armin Rauschenberger, Lucie Remark, Antonio Rodriguez, Basile Rommes, Kirsten Rump, Estelle Sandt, Bruno Santos, Venkata P. Satagopam, Aurélie Sausy, Margaux Schmitt, Christiane Schmitt, Reinhard Schneider, Valerie Schröder, Serge Schumacher, Alexandra Schweicher, Sneeha Seal, Jean-Yves Servais, Florian Simon, Amna Skrozic, Chantal Snoeck, Kate Sokolowska, Lara Stute, Hermann Thien, Stéphane Toll, Noua Toukourou, Christophe Trefois, Johanna Trouet, Nguyen Trung1, Jonathan Turner, Michel Vaillant, Daniela Valoura Esteves, Carlos Vega Moreno, Charlène Verschueren, Maharshi Vyas, Claus Vögele, Cécile Walczak, Xinhui Wang, Femke Wauters, Bernard Weber, Emilie Weibel, Tania Zamboni

**KoCo19 study group**

Mohamed Ibraheem Mohamed Ahmed, Emad Alamoudi, Jared Anderson, Valeria Baldassarre, Abhishek Bakuli, Maximilian Baumann, Marc Becker, Franziska Bednarski, Marieke Behlen, Olimbek Bemirayev, Jessica Beyerl, Patrick Bitzer, Rebecca Böhnlein, Isabel Brand, Anna Brauer, Vera Britz, Jan Bruger, Franziska Bünz, Friedrich Caroli, Josephine Coleman, Lorenzo Contento, Alina Czwienzek, Flora Deák, Maximilian N. Diefenbach, Jana Diekmannshemke, Paulina Diepers, Anna Do, Gerhard Dobler, Jürgen Durner, Tabea Eser, Ute Eberle, Judith Eckstein, Philine Falk, Heike Fensterseifer, Manuela Feyereisen, Volker Fingerle, Stefanie Fischer, Jonathan Frese, Felix Forster, Günter Fröschl, Christiane Fuchs, Otto Geisenberger, Mercè Garí, Marius Gasser, Sonja Gauder, Raffaela Geier, Kristina Gillig, Christof Geldmacher, Keisha Gezgin, Leonard Gilberg, Kristina Gillig, Philipp Girl, Elias Golschan, Vitus Grauvogl, Jessica Michelle Guggenbuehl Noller, Elena Maria Guglielmini, Pablo Gutierrez, Anselm Haderer, Celina Halfmann, Marlene Hannes, Lena Hartinger, Timm Haselwarter, Jan Hasenauer, Alejandra Hernandez, Luca Heller, Arlett Heiber, Matthias Herrmann, Leah Hillari, Stefan Hillmann, Christian Hinske, Janna Hoefflin, Tim Hofberger, Michael Höfinger, Larissa Hofmann, Sacha Horn, Kristina Huber, Christian Janke, Lilian Karger, Ursula Kappl, Antonia Keßler, Zohaib Khan, Charlotte Kiani, Isabel Klugherz, Norah Kreider, Johanna Kresin, Arne Kroidl, Pratik Kunder, Magdalena Lang, Clemens Lang, Silvan Lange, Ekaterina Lapteva, Michael Laxy, Ronan Le Gleut, Reiner Leidl, Leopold Liedl, Felix Lindner, Xhovana Lucaj, Elisabeth Lucke, Fabian Luppa, Alexandra Sophie Nafziger, Alexander Maczka, Petra Mang, Alisa Markgraf, Paula Matcau, Rebecca Mayrhofer, Anna-Maria Mekota, Dafni Metaxa, Emily Mohr, Hannah Müller, Katharina Müller, Nathalia Nascimento, Kasimir Niermeyer, Sophia Nikolaides, Ivan Noreña, Laura Olbrich. Leonie Pattard, Ivana Paunovic, Michael Plank, Claire Pleimelding, Michel Pletschette, Viona Poll, Michael Pritsch, Stephan Prückner, Kerstin Puchinger, Konstantin Pusl, Peter Pütz, Katja Radon, Elba Raimúndez, Julius Raschka, Jakob Reich, Friedrich Riess, Camilla Rothe, Raquel Rubio-Acero, Viktoria Ruci, Elmar Saathoff, Nicole Schäfer, Yannik Schälte, Paul Schandelmaier, Benedikt Schluse, Annika Schneider, Lara Schneider, Sophie Schultz, Mirjam Schunk, Lars Schwettmann, Josefin Sedlmeier, Linda Sintu-Sempta, Alba Soler, Peter Sothmann, Katharina Strobl, Aida Strüber, Laura Strüber, Jeni Tang, Fabian Theis, Verena Thiel, Angelika Thomschke, Eva Thumser, Niklas Thur, Sophie Thiesbrummel, Julian Ullrich, Vincent Vollmayr, Emilia Von Lovenberg, Jonathan Von Lovenberg, Carsten Vos, Julia Waibel, Claudia Wallrauch, Nikolas Weigl, Simon Winter, Roman Wölfl, Julia Wolff, Pia Wullinger, Tobias Würfel, Patrick Wustrow, Sabine Zange, Eleftheria Zeggini, Anna Zielke, Thorbjörn Zimmer, Thomas Zimmermann, Lea Zuche.

**EpiCov study group**

Josiane Warszawski, Nathalie Bajos, Guillaume Bagein, François Beck, Emilie Counil, Florence Jusot, Nathalie Lydié, Claude Martin, Laurence Meyer, Philippe Raynaud, Alexandra Rouquette, Ariane Pailhé, Delphine Rahib, Patrick Sillard, Alexis Spire.
