# Supplementary material for: Waning of anti-SARS-CoV-2 antibodies after the first wave of the COVID-19 pandemic in 2020: A 12-month-evaluation in three population-based European studies
Source: PLoS One. 2025 May 9;20(5):e0320196. doi: 10.1371/journal.pone.0320196 (PMC12063904; doi:10.1371/journal.pone.0320196)
Supplement: Attachment — Submitted filename: WA_PlosOne_Response to review_1.0.docx [file pone.0320196.s005.docx]

# Journal Requirements:

1. When submitting your revision, we need you to address these additional requirements.

Please ensure that your manuscript meets PLOS ONE's style requirements, including those for file naming. The PLOS ONE style templates can be found at

<https://journals.plos.org/plosone/s/file?id=wjVg/PLOSOne_formatting_sample_main_body.pdf>

and
<https://journals.plos.org/plosone/s/file?id=ba62/PLOSOne_formatting_sample_title_authors_affiliations.pdf>

Response: We have formatted the manuscript according to style requirements.

2. Thank you for stating the following in the Competing Interests section: 
I have read the journal's policy and the authors of this manuscript have the following competing interests: 
A.W. and M.H. report personal fees and non-financial support from Roche Diagnostics. A.W. and M.H. report non-financial support from Euroimmun, non-financial support from Viramed, non-financial support from Mikrogen. A.W. and M.H. report grants, non-financial support and other from German Centre for Infection Research DZIF, grants and non-financial support from Government of Bavaria, non-financial support from BMW, non-financial support from Munich Police, nonfinancial support and other from Accenture. A.W. and M.H. report personal fees and nonfinancial support from Dr. Box-Betrobox, non-financial support from Dr. Becker MVZ during the conduct of the study. A.W. is involved in other different patents and companies not in relation to the serology of SARS-CoV-2. A.W. reports personal fees and others from Haeraeus Sensors, nonfinancial support from Bruker Daltonics, all of which are outside the submitted work, and non-related to SARS-CoV-2. The other authors declare that they have no competing interests. The funders had no role in the design of the study; in the collection, analyses, or interpretation of data; in the writing of the manuscript; or in the decision to publish the results.
   
We note that one or more of the authors are employed by a commercial company.

a. Please provide an amended Funding Statement declaring this commercial affiliation, as well as a statement regarding the Role of Funders in your study. If the funding organization did not play a role in the study design, data collection and analysis, decision to publish, or preparation of the manuscript and only provided financial support in the form of authors' salaries and/or research materials, please review your statements relating to the author contributions, and ensure you have specifically and accurately indicated the role(s) that these authors had in your study. You can update author roles in the Author Contributions section of the online submission form.

Please also include the following statement within your amended Funding Statement. 
“The funder provided support in the form of salaries for authors, but did not have any additional role in the study design, data collection and analysis, decision to publish, or preparation of the manuscript. The specific roles of these authors are articulated in the ‘author contributions’ section.”
If your commercial affiliation did play a role in your study, please state and explain this role within your updated Funding Statement.

Response: We have amended the Funding Statement accordingly.

b. Please also provide an updated Competing Interests Statement declaring this commercial affiliation along with any other relevant declarations relating to employment, consultancy, patents, products in development, or marketed products, etc.

Within your Competing Interests Statement, please confirm that this commercial affiliation does not alter your adherence to all PLOS ONE policies on sharing data and materials by including the following statement: "This does not alter our adherence to  PLOS ONE policies on sharing data and materials.” (as detailed online in our guide for authors http://journals.plos.org/plosone/s/competing-interests) . If this adherence statement is not accurate and  there are restrictions on sharing of data and/or materials, please state these. Please note that we cannot proceed with consideration of your article until this information has been declared.

Please include both an updated Funding Statement and Competing Interests Statement in your cover letter. We will change the online submission form on your behalf.

Response: We have included an updated Funding Statement and Competing Interests Statement in the cover letter. Regarding data availability, please see our response below.

3. In the online submission form, you indicated that Koco19: The data that support the findings of this study are available from the KoCo19 collaboration group, but restrictions apply to the availability of these data, which were used under license for the current study, and so are not publicly available. Data are however available from the authors upon reasonable request and with permission of the KoCo19 collaboration group.

EpiCov: The second round EpiCov dataset is available for research purpose on CASD (https://www.casd.eu/), after submission to approval of French Ethics and Regulatory Committee procedure (Comité du Secret Statistique, CESREES and CNIL). Access to anonymized individual data underlying the findings may be available before the planned period, on request to the corresponding author, to be submitted to approval of ethics and reglementary Committee for researchers who meet the criteria for access to data.

CON-VINCE: The dataset for this manuscript is not publicly available as it is linked to the CON-VINCE Study and its internal regulations. Any requests for accessing the dataset can be directed at con-vince@lih.lu. All data of the manuscript will be provided upon reasonable request and approval by the ethics committee.

All PLOS journals now require all data underlying the findings described in their manuscript to be freely available to other researchers, either a. In a public repository, b. Within the manuscript itself, or c. Uploaded as supplementary information.

This policy applies to all data except where public deposition would breach compliance with the protocol approved by your research ethics board. If your data cannot be made publicly available for ethical or legal reasons (e.g., public availability would compromise patient privacy), please explain your reasons on resubmission and your exemption request will be escalated for approval.

Response: This study operates under General Data Protection Regulation (GDPR) and Germany, France and Luxembourg data protection laws. The consent forms emphasized implementing appropriate security measures and strictly confidential handling. In addition, data transfers outside the EU require appropriate measures and safeguards.

Public release would bypass these transfer control mechanisms.

That is why, access to data is possible only by the request procedures that were put in place and approved by the authorities and ethics board of each country, previously described.

4. One of the noted authors is a group or consortium "the ORCHESTRA working group". In addition to naming the author group, please list the individual authors and affiliations within this group in the acknowledgments section of your manuscript. Please also indicate clearly a lead author for this group along with a contact email address.

Response: We now have listed the individual authors and affiliations within the ORCHESTRA working group in the acknowledgments section of your manuscript, instead of in an Appendix. We have now indicated Laurence Meyer as the lead author of the ORCHESTRA working group.

5. Please include captions for your Supporting Information files at the end of your manuscript, and update any in-text citations to match accordingly. Please see our Supporting Information guidelines for more information: http://journals.plos.org/plosone/s/supporting-information.

Response: We have edited the manuscript accordingly

**Reviewers' comments:**

**Reviewer's Responses to Questions**

Comments to the Author

1. Is the manuscript technically sound, and do the data support the conclusions?

The manuscript must describe a technically sound piece of scientific research with data that supports the conclusions. Experiments must have been conducted rigorously, with appropriate controls, replication, and sample sizes. The conclusions must be drawn appropriately based on the data presented.

Reviewer #1: Partly

Reviewer #2: Yes

2. Has the statistical analysis been performed appropriately and rigorously?

Reviewer #1: Yes

Reviewer #2: Yes

3. Have the authors made all data underlying the findings in their manuscript fully available?

The [PLOS Data policy](http://track.editorialmanager.com/CL0/http:%2F%2Fwww.plosone.org%2Fstatic%2Fpolicies.action%23sharing/1/010f0192d42d1361-5e782444-d358-4fe0-bf90-a38fd32ff33a-000000/By9qQ9aDCsDXvHMQtKIFmSjpz7TM9EMCZSGmlHECYjI=182) requires authors to make all data underlying the findings described in their manuscript fully available without restriction, with rare exception (please refer to the Data Availability Statement in the manuscript PDF file). The data should be provided as part of the manuscript or its supporting information, or deposited to a public repository. For example, in addition to summary statistics, the data points behind means, medians and variance measures should be available. If there are restrictions on publicly sharing data—e.g. participant privacy or use of data from a third party—those must be specified.

Reviewer #1: No

Reviewer #2: Yes

4. Is the manuscript presented in an intelligible fashion and written in standard English?

PLOS ONE does not copyedit accepted manuscripts, so the language in submitted articles must be clear, correct, and unambiguous. Any typographical or grammatical errors should be corrected at revision, so please note any specific errors here.

Reviewer #1: Yes

Reviewer #2: Yes

5. Review Comments to the Author

Please use the space provided to explain your answers to the questions above. You may also include additional comments for the author, including concerns about dual publication, research ethics, or publication ethics. (Please upload your review as an attachment if it exceeds 20,000 characters)

# Reviewer #1:

The manuscript by Dr Novelli and colleagues aimed to study the evolution of IgG antibodies to SARS-CoV2 Spike antigen over time. To that end, the authors used three cohorts, from Germany, France and Luxemburg, established in the first half of the year 2020 in the three countries. For their study, they included a subpopulation in each of the three cohorts and tested selected plasma samples with an ELISA assay allowing the detection of IgG to SARS-CoV2 S protein.

After testing 65, 599 and 16 samples of the cohorts from Germany, France and Luxemburg, respectively, the authors observed no sero-negativation four months after infection and then a reversion in roughly 1/3 of the patients 6 and 12 months after infection. The authors concluded on the waning of IgG antibodies 6 to 12 months after primary infection in one third of infected persons in their cohorts.

The authors addressed here an important issue in the immunity to SARS-CoV2 and its duration. This type of study might also help address the question of herd immunity. The authors are commended for that. The work presents also limitations, some of which mentioned by the authors in the discussion. These include:

1. The role of re-infection in the maintenance of IgG antibody level. This point is important and should be discussed further, especially in the light of what is known on covid-19 in the three countries during the study period.

Response: A limit of our work is that we are not able to assess possible reinfections between IgG measurements. This could be a concern for measurements at 6 and 12 months in the EpiCov and CON-VINCE cohort, respectively. As reinfections induce a rise in antibody production, this could have result in underestimating IgG waning.

However, during the period from July to November 2020, there was no significant antigenic change in the SARS-CoV-2 strains circulating in France (Ding et al. 2022; Wu et al. 2022). The “European” variant D614G probably began circulating in January 2020, and the alpha variant was detected in the United Kingdom in September 2020, and only in December 2020 in France. Furthermore, given the low prevalence in the population at that time, the evolution of the virus was not guided by immunological selection forces, but by forces leading to a gradual increase in transmission. Hence, there is little chance that immunocompetent individuals infected at the start of circulation of the D614G variant in France would have been reinfected within a few weeks by the alpha variant.

Moreover, protective efficacy of previous exposure against viral variants was reported to be very high for variants that circulated at this time of the pandemic (Helfand et al. 2022; Smolenov et al. 2022). A meta-analysis in the setting of the wild-type and the Alpha variant reported that reinfection was an uncommon event (range, 0% to 2.2%).

To complete our results, we also performed additional analyses on samples from EpiCov participants. We measured anti-N IgG levels 6 months after enrolment (November 2020) using Anti-SARS-CoV-2 NCP ELISA kits (Euroimmun®, Lübeck, Germany) for the detection of anti-nucleocapsid SARS-CoV-2 antibodies. As anti-N antibodies have shorter half-life than anti-S antibodies, they are considered in some settings to be marker of recent infection.

Among the 523 participants in whom anti-N IgG levels could have been measured in November-December 2020, 29% (155/523) had positive anti-N ELISA serology. Among these 155 participants, levels of anti-N OD ratio were moderate (median (IQR) 1.56 (1.29 – 2.17)). Only a limited number of participants (n=20) showed “high” anti-N OD ratios (>3) and were associated with higher albeit modest IgG anti-S OD ratio (median (IQR) 4.23 (2.48 – 5.19)) compared to the overall population of anti-S positive samples (mean 2.46 (1.15 – 3.82)). Such distribution was consistent with the presence of residual anti-N antibodies produced after the original infection.

Moreover, anti-N positivity in November 2020 was associated with higher neutralizing antibody titers in May 2020 (see Table below). This is in favour of individuals producing a strong antibody response after infection, still detectable 6 months later.

Although, we cannot completely rule out the possibility of few individuals may have been reinfected, the overall picture is rather that of a “classic” gradual decrease in antibodies over the fairly short period between May and November 2020.

**Table. ELISA anti-N IgG OD ratios in November 2020 and Neutralizing antibodies in May 2020**

|  | **Anti-N IgG in November 2020** | | |  |
| --- | --- | --- | --- | --- |
| **Neutralizing antibodies in May 2020** | **Negative**  **N = 351** | **Intermediate**  **N = 17** | **Positive**  **N = 155** | **P-value** |
| **Negative** (VNT < 40) | 31.2 (105/337) | 41.2 (7/17) | 12.2 (18/147) | < 0.001 |
| **Positive** (VNT ≥ 40) | 68.8 (232/337) | 58.8 (10/17) | 87.8 (129/147) |  |
| Missing | 14 | 0 | 8 |  |

% (n/N)

Fisher’s exact test

We added these elements and discussed these additional results to the manuscript as follows:

Methods/Laboratory analyses (lines 171 – 173):

” Samples from the 2nd round for which sufficient material remained for additional analyses were analyzed for the detection of antibodies (IgG) against the N-protein of SARS-CoV-2 (Euroimmun®, Lübeck, Germany).”

Methods/Statistical analyses (lines 223 – 225):

“To investigate whether the persistence of anti-S antibodies could be partially explained by the occurrence of reinfections, anti-N IgG levels were also described 6 months after enrolment in the EpiCov cohort, as a marker of recent infection”.

Results/ Proportion of subsequent SARS-Cov-2 seroreversion (lines 283 – 291):

“To investigate whether the persistence of anti-S antibodies could be partially explained by occurrence of reinfection, we measured anti-N IgG levels in samples from 523 EpiCov participants in November 2020. Overall, 29% (155/523) had positive anti-N ELISA serology 6 months after enrolment, with a median OD ratio of 1.56 (IQR, 1.29 – 2.17). Among the participants who remained positive for anti-S antibodies, 45% had positive anti-N IgG levels (see S1 Table). Only a limited number of participants (n=20) showed anti-N OD ratios (>3) and were associated with higher albeit modest IgG anti-S OD ratio (median (IQR) 4.23 (2.48 – 5.19)) compared to the overall population of anti-S positive samples (mean 2.46 (1.15 – 3.82)). Anti-N positivity in November 2020 was associated with higher neutralizing antibody titres in May 2020 (see S2 Table).”

Discussion (lines 382 – 393)

“We were not able to assess the incidence of reinfections between IgG measurements. However, several arguments suggest this was uncommon event during the study period. First, the antigenic closeness and protective efficacy confer by the exposure to the variants circulating over the study period i.e., the D614G variants and then Alpha variant from the end of December 2020 (41–44) makes reinfection unlikely. To go further, we measured in EpiCov participants anti-N antibodies at 6 months after enrolment, which, given their shorter half-life than anti-S antibodies, could be considered markers of recent infections. Six months after enrolment, participants showed low or moderate anti-N IgG levels, even those with positive ELISA-S serology, consistent with a gradual decrease in antibodies over the fairly short period between May and November 2020, rather than recent infection. Besides, the association of anti-N IgG positivity in November 2020 with seroprotection in May 2020, as shown by neutralizing antibody titers, in May 2020 is in favour of individuals producing a strong antibody response after infection, still detectable 6 months later.”

*References*

41. Ding LS, Zhang Y, Wen D, Ma J, Yuan H, Li H, et al. Growth, Antigenicity, and Immunogenicity of SARS-CoV-2 Spike Variants Revealed by a Live rVSV-SARS-CoV-2 Virus. Front Med [Internet]. 7 janv 2022 [cité 15 janv 2025];8. Disponible sur: https://www.frontiersin.org/journals/medicine/articles/10.3389/fmed.2021.793437/full

42. Wu J, Nie J, Zhang L, Song H, An Y, Liang Z, et al. The antigenicity of SARS-CoV-2 Delta variants aggregated 10 high-frequency mutations in RBD has not changed sufficiently to replace the current vaccine strain. Signal Transduct Target Ther. 19 janv 2022;7(1):1‑10.

43. Helfand M, Fiordalisi C, Wiedrick J, Ramsey KL, Armstrong C, Gean E, et al. Risk for Reinfection After SARS-CoV-2: A Living, Rapid Review for American College of Physicians Practice Points on the Role of the Antibody Response in Conferring Immunity Following SARS-CoV-2 Infection. Ann Intern Med. 19 avr 2022;175(4):547‑55.

44. Smolenov I, Han HH, Li P, Baccarini C, Verhoeven C, Rockhold F, et al. Impact of previous exposure to SARS-CoV-2 and of S-Trimer (SCB-2019) COVID-19 vaccination on the risk of reinfection: a randomised, double-blinded, placebo-controlled, phase 2 and 3 trial. Lancet Infect Dis. 1 juill 2022;22(7):990‑1001.

2. Although the 3 countries are neighbors, political decisions during the early phase of covid-19 were not exactly the same and this might have some incidence on epidemiological situation in the three countries. This should be mentioned in the manuscript.

Response: We agree with the reviewer. We have added the following sentences in the discussion (lines 406 – 410):

“We present results from three neighboring countries that experienced COVID-19 waves at the same time, and made close decisions to reduce transmission of the virus. Although the political decisions taken to manage the pandemic were not exactly the same, France being the country most affected and with the most restrictive measures, this contributes greatly to the comparability of the three cohorts.”

3. The initial cohorts from the three countries included large sample size. However, in the studied population, the sample size per site is very disequilibrated (16, 65 and 599). Did the authors compare these 3 populations? How comparable were they? If they were not comparable, then it is very risky to draw a general conclusion on antibody dynamics from 3 different population in size and structure.

Response: The three cohorts Koco19, EpiCov, and CON-VINCE have in common to be Western European, population-based and launched at the same period of the COVID-19 pandemic, and IgG were measured with the same technique.

We agree with the reviewer that the question of comparability is important. that they are not entirely comparable. EpiCov and CON-VINCE are nationwide cohorts, while Koco19 is regional.

For these reasons, we presented the results separately for each cohort and stratified by age and sex.

We emphasized more this point in the Discussion as follows (lines 410-412):

“However, each cohort has its own regional or national scope, which explains the differences in sample size and sampling schedules. We took advantage of these differences by showing seroconversion rates separately in each cohort and at different timepoints.”

4. Can the authors indicate the sensitivity and specificity of the ELISA assay used?

Response: We added this information in the Methods\Laboratory analyses section (lines 156-158):

“The Euroimmun ELISA-S test has a sensitivity of 94.4%, according to the manufacturer’s cutoff. It has been evaluated in various studies, which reported a specificity ranging from 96.2 to 100% and sensitivity ranging from 86.4 to 100% (29–32).”

*References*

29. Beavis KG, Matushek SM, Abeleda APF, Bethel C, Hunt C, Gillen S, et al. Evaluation of the EUROIMMUN Anti-SARS-CoV-2 ELISA Assay for detection of IgA and IgG antibodies. J Clin Virol. 2020;129:104468. doi: 10.1016/j.jcv.2020.104468.

30. Inchauste L, Nurtop E, Brisbarre N, Ninove L, Gallian P, de Lamballerie X, et al. Exploring cell-free assays for COVID-19 serosurvey. Sci Rep. 13 mars 2024;14(1):609

31. Kohmer N, Westhaus S, Rühl C, Ciesek S, Rabenau HF. Clinical performance of different SARS-CoV-2 IgG antibody tests. J Med Virol. 2020;92(10):2243–2247. doi: 10.1002/jmv.26145

32. Krüttgen A, Cornelissen CG, Dreher M, Hornef M, Imöhl M, Kleines M. Comparison of four new commercial serologic assays for determination of SARS-CoV-2 IgG. J Clin Virol. 2020;128:104394. doi: 10.1016/j.jcv.2020.104394.

5. The authors defined two categories in the ELISA results according to OD ratios: below 0.7, the samples were considered negative and above 1.1, they were considered positive. What about those in between? It is written in the manuscript that samples with OD ration above 0.7 were tested for neutralization. How many of those with OD ratio between 0.7 and 1.1 were positive in neutralization (i.e. as per authors’ definition with a titer above 40)? This should be helpful to readers and should be presented.

Response: Among the 814 participants with an ELISA serology in May 2020, 241 participants had OD ratios between 0.7 and 1.1, of which 38% (92/241) were positive for neutralizing antibodies.

The percentage of participants positive for seroneutralisation was 71% among ELISA positive (OD ratio > 1.1) participants. This percentage fell to 36% among participants with an intermediate result (0.7-1.1). See table below.

|  | **Neutralizing antibodies** | | | **Total** |
| --- | --- | --- | --- | --- |
| **Anti-S ELISA OD ratio** | **Missing** | **Negative**  **VNT <40** | **Positive**  **VNT ≥40** |  |
| **< 0.7** | 7564 | - | - | 7564 |
| **0.7 – 1.1** | 14 | 149 | 92 | 255 |
| **>1.1** | 26 | 146 | 427 | 599 |

A positive ELISA test (OD ratio > 1.1) was considered as a specific marker of past infection, while the interpretation of intermediate results should be interpreted more cautiously. The intermediate group (OD ratio between 0.7 and 1.1) may mix “previously infected” and “naïve to infection” participants who cannot be distinguished by serological analysis.

We have emphasized this point further in the discussion (lines 354 -360):

“We applied strong selection criteria by including only participants with positive anti-S IgG OD ratio> 1.1 in May 2020, and defined seroreversion as achieving an OD ratio < 0.7 over time. In this way, we aimed to be specific in defining seroreversion. We may have omitted people who were infected at the very start of the pandemic and whose IgG OD ratio was already below 1.1 in May 2020. Our results may therefore underestimate antibody seroreversion rate, and should be interpreted as a minimum estimate of seroreversion in the general population.”

# Reviewer #2:

Waning of anti-SARS-CoV-2 antibodies after the first wave of the COVID-19 pandemic in 2020: a 12-month-evaluation in three population-based European studies.

In this study, the decrease in antibody levels in unvaccinated Anti-S IgG seropositive individuals from three different centers in Europe was investigated as a retrospective cohort study. The inclusion of Anti-S IgG reactive individuals in the early phase of the COVID-19 Pandemic (in 2020) may be valuable in terms of understanding the effect of natural infection on antibody kinetics in the absence of the vaccine factor. It is appropriate to publish after the necessary major and minor revisions.

Major
1- The principled test used for the virus neutralization test (VNT) should be specified in the Material - Method section. In this context, PRNT (Plaque Reduction Neutralization Test) or Surrogate Neutralization Tests can be used [1].

• In addition, the diagnostic reference values of the neutralization test results should be specified according to the relevant method used (positive and negative limit values).

Response: We added precision about the virus neutralization test in the Methods/Laboratory analyses section (lines 160-166).

“All samples with an ELISA-S test optical density ratio ≥ 0.7 were tested with an in-house microneutralization assay to detect neutralizing anti-SARS-CoV-2 antibodies. For this assay, TMPRSS2-expressing VeroE6 cells cultured in 96-well microplates, 100 TCID50 of the SARS-CoV-2 strain BavPat1 (courtesy of Prof. Drosten, Berlin, Germany) and serial dilutions of serum (1/20–1/160) were used, as described elsewhere (Gallian et al. 2020). Dilutions associated with the presence or absence of a cytopathic effect on 4.5 days after infection were considered negative and positive, respectively. The virus neutralization titer (VNT) referred to the highest dilution of serum with a positive result. Specimens with a VNT ≥ 40 were considered positive, as the specificity at this threshold was 100% on 486 samples collected before the emergence of SARS-CoV-2 in 2017.”

*References*

Gallian P, Pastorino B, Morel P, Chiaroni J, Ninove L, de Lamballerie X. Lower prevalence of antibodies neutralizing SARS-CoV-2 in group O French blood donors. Antivir Res. 2020;181:104880. doi: 10.1016/j.antiviral.2020.104880.

2- Has a power analysis been performed to support the sample power? If so, it should be stated in the Material-Method section.

Response: No power analysis was performed. We included all eligible participants from the 3 preexisting European cohorts.

3- The variant that was effective at the beginning of the COVID-19 pandemic was D614G. Later, the Alpha (B.1.1.7.) variant was effective. In this context, the development of reinfection in the cases included in the study should be taken into consideration and explained.

Response: We agree with the reviewer. We propose to add the following elements to the manuscript (see response 1 to reviewer 1).

Methods/Laboratory analyses (lines 171 – 173):

“Samples from the 2nd round for which sufficient material remained for additional analyses were analyzed for the detection of antibodies (IgG) against the N-protein of SARS-CoV-2 (Euroimmun®, Lübeck, Germany).”

Methods/Statistical analyses (lines 223 – 225):

“To investigate whether the persistence of anti-S antibodies could be partially explained by the occurrence of reinfections, anti-N IgG levels were also described 6 months after enrolment in the EpiCov cohort, as a marker of recent infection”.

Results/ Proportion of subsequent SARS-Cov-2 seroreversion (lines 283 – 291):

“To investigate whether the persistence of anti-S antibodies could be partially explained by occurrence of reinfection, we measured anti-N IgG levels in samples from 523 EpiCov participants in November 2020. Overall, 29% (155/523) had positive anti-N ELISA serology 6 months after enrolment, with a median OD ratio of 1.56 (IQR, 1.29 – 2.17). Among the participants who remained positive for anti-S antibodies, 45% had positive anti-N IgG levels (see S1 Table). Only a limited number of participants (n=20) showed anti-N OD ratios (>3) and were associated with higher albeit modest IgG anti-S OD ratio (median (IQR) 4.23 (2.48 – 5.19)) compared to the overall population of anti-S positive samples (mean 2.46 (1.15 – 3.82)). Anti-N positivity in November 2020 was associated with higher neutralizing antibody titres in May 2020 (see S2 Table).”

Discussion (lines 382 – 393)

“We were not able to assess the incidence of reinfections between IgG measurements. However, several arguments suggest this was uncommon event during the study period. First, the antigenic closeness and protective efficacy confer by the exposure to the variants circulating over the study period i.e., the D614G variants and then Alpha variant from the end of December 2020 (41–44) makes reinfection unlikely. To go further, we measured in EpiCov participants anti-N antibodies at 6 months after enrolment, which, given their shorter half-life than anti-S antibodies, could be considered markers of recent infections. Six months after enrolment, participants showed low or moderate anti-N IgG levels, even those with positive ELISA-S serology, consistent with a gradual decrease in antibodies over the fairly short period between May and November 2020, rather than recent infection. Besides, the association of anti-N IgG positivity in November 2020 with seroprotection in May 2020, as shown by neutralizing antibody titers, in May 2020 is in favour of individuals producing a strong antibody response after infection, still detectable 6 months later.”

*References*

41. Ding LS, Zhang Y, Wen D, Ma J, Yuan H, Li H, et al. Growth, Antigenicity, and Immunogenicity of SARS-CoV-2 Spike Variants Revealed by a Live rVSV-SARS-CoV-2 Virus. Front Med [Internet]. 7 janv 2022 [cité 15 janv 2025];8. Disponible sur: https://www.frontiersin.org/journals/medicine/articles/10.3389/fmed.2021.793437/full

42. Wu J, Nie J, Zhang L, Song H, An Y, Liang Z, et al. The antigenicity of SARS-CoV-2 Delta variants aggregated 10 high-frequency mutations in RBD has not changed sufficiently to replace the current vaccine strain. Signal Transduct Target Ther. 19 janv 2022;7(1):1‑10.

43. Helfand M, Fiordalisi C, Wiedrick J, Ramsey KL, Armstrong C, Gean E, et al. Risk for Reinfection After SARS-CoV-2: A Living, Rapid Review for American College of Physicians Practice Points on the Role of the Antibody Response in Conferring Immunity Following SARS-CoV-2 Infection. Ann Intern Med. 19 avr 2022;175(4):547‑55.

44. Smolenov I, Han HH, Li P, Baccarini C, Verhoeven C, Rockhold F, et al. Impact of previous exposure to SARS-CoV-2 and of S-Trimer (SCB-2019) COVID-19 vaccination on the risk of reinfection: a randomised, double-blinded, placebo-controlled, phase 2 and 3 trial. Lancet Infect Dis. 1 juill 2022;22(7):990‑1001.

4- The reason why Anti-N (Nucleocapsid-specific antibody), which is an important antibody in the post-infection situation, is not preferred instead of Anti-S measurement after SARS-CoV-2 infection should be explained.

Response: Koco19, EpiCov and CON-VINCE are three cohorts that were initially launched at the start of the May 2020 pandemic to track the spread of SARS-CoV-2 infection in the general population. In this study, we took advantage of repeated anti-S IgG measurements to estimate the waning of antibodies over time.

We acknowledge that the measurement of anti-N antibodies is also of great interest, particularly for documenting reinfections. Following the two reviewers' comments, we have also measured anti-N antibodies in EpiCov participants. (See also response to reviewer 1, Q1 and reviewer 2, Q3).

5- In the results of the study, it was stated that seroreversion was observed more frequently in the 30-39 age group compared to the 15-29 age group and that it was statistically significant (p<0.001). At this point, it was stated in the discussion section that the 30-39 age group is more socioactive as a possible reason underlying the more frequent seronegativity observed compared to the older age group. This explanation may be possible due to the possibility of early infection. However, in line with basic immunological phenomena, there is more antibody production in young individuals [2, 3]. For example, while 4000 IU/mL of Antibody can be formed in a young individual, 2000 IU/mL of antibody can be formed in an older individual. In this case, it is interesting that a young individual may have early infection but falls into seronegativity earlier or more frequently than an older individual within the elapsed time period. This situation should be clarified from an immunological perspective and further explanations should be provided.

Response: We agree with the reviewer. This is a paradoxical result that we cannot fully explain.

We observed the highest percentage (54.7%) of seroreversion in participants aged 30-39, whereas we expected the highest seroconversion rates in older participants, who are known to produce fewer antibodies.

The 30-39 age group was also the group with the lowest IgG levels at baseline in May 2020 (see table below, EpiCov cohort).

|  | **IgG anti-Q OD Ratio in May 2020** | | | | | |
| --- | --- | --- | --- | --- | --- | --- |
| **Age, years** | **Min** | **Q1** | **Mean** | **Median** | **Q3** | **Max** |
| **15-29** | 0.83 | 1.14 | 2.84 | 2.95 | 3.94 | 8.39 |
| **30-39** | 0.80 | 1.13 | 1.99 | 1.76 | 2.20 | 7.79 |
| **40-49** | 0.81 | 1.02 | 2.23 | 1.50 | 3.01 | 9.91 |
| **50-59** | 0.80 | 1.71 | 4.05 | 3.41 | 6.02 | 10.52 |
| **≥60** | 0.80 | 0.87 | 2.39 | 1.00 | 2.41 | 10.96 |

Several factors may explain this result. Firstly, antibody levels may be associated with symptom severity (Röltgen et al. 2020; Yan et al. 2022), and younger people are known to have less severe or less symptomatic forms. Second, as noted in the manuscript, we hypothesized that 30–39-year-olds were infected earlier in the pandemic and had already lost some antibodies by the time of EpiCov enrollment in May 2020. As we only selected participants with an OD ratio > 1.1 in May 2020, it is possible that we exert differential selection pressure by age group, which could contribute to this unexpected result.

Therefore, we have discussed more this point as follows (lines 366-374):

“Of note, the highest seroreversion rate (54%) was observed in participants aged 30-39, although this result was no longer statistically significant in the multivariable analysis. This is an unexpected result as young people are usually known to show higher and more persistent antibodies levels than older people. However, in our study, the 30-39 age group already had the lowest IgG levels at enrolment, in May 2020. Since antibody levels are associated with symptom severity (Röltgen et al. 2020; Yan et al. 2022), and younger people had fewer symptoms, their initial antibody production may not have been so high compared with other age groups. Secondly, 30–39-year-olds may have been infected earlier in the pandemic and had already lost some antibodies by the time they were enrolled in EpiCov in May 2020.”

*References*

Röltgen K, Powell AE, Wirz OF, Stevens BA, Hogan CA, Najeeb J, et al. Defining the features and duration of antibody responses to SARS-CoV-2 infection associated with disease severity and outcome. Sci Immunol 2020;5:eabe0240. https://doi.org/10.1126/sciimmunol.abe0240.

Yan X, Chen G, Jin Z, Zhang Z, Zhang B, He J, et al. Anti‐SARS‐CoV‐2 IgG levels in relation to disease severity of COVID‐19. J Med Virol 2022;94:380–3. <https://doi.org/10.1002/jmv.27274>

6- As a result, seroreversion was investigated in the cases included in the study in line with decreasing antibodies. Although scientific inferences have been examined very well at this point, recommendations should also be shared in terms of public health. What is recommended for individuals who are seronegative within a certain period of time?

Response: Our work has no direct bearing on clinical practice and recommendations, especially in the current era of vaccination, but it provides useful information that need to be accounted for when inferring incidence from data from population-based repeated seroprevalence surveys

**Minor**
1- Virus neutralization test principle and commercial kit identification, if used, should be provided.

Response: We added precision about the virus neutralization test in the Methods\Laboratory analyses section (lines 162-170).

“All samples with an ELISA-S test optical density ratio ≥ 0.7 were tested with an in-house microneutralization assay to detect neutralizing anti-SARS-CoV-2 antibodies. For this assay, TMPRSS2-expressing VeroE6 cells cultured in 96-well microplates, 100 TCID50 of the SARS-CoV-2 strain BavPat1 (courtesy of Prof. Drosten, Berlin, Germany) and serial dilutions of serum (1/20–1/160) were used, as described elsewhere [[1](https://pmc.ncbi.nlm.nih.gov/articles/PMC8743062/#CR6)]. Dilutions associated with the presence or absence of a cytopathic effect on 4.5 days after infection were considered negative and positive, respectively. The virus neutralization titer (VNT) referred to the highest dilution of serum with a positive result. Specimens with a VNT ≥ 40 were considered positive, as the specificity at this threshold was 100% on 486 samples collect before the emergence of SARS-Cov-2 in 2017.”

*References*

Gallian P, Pastorino B, Morel P, Chiaroni J, Ninove L, de Lamballerie X. Lower prevalence of antibodies neutralizing SARS-CoV-2 in group O French blood donors. Antivir Res. 2020;181:104880. doi: 10.1016/j.antiviral.2020.104880.

References
1- Dinç, H. Ö., Demirci, M., Özdemir, Y. E., Sirekbasan, S., Aktaş, A. N., Karaali, R., ... & Kocazeybek, B. (2022). Anti-SARS-CoV-2 IgG and neutralizing antibody levels in patients with past COVID-19 infection: a longitudinal study. Balkan Medical Journal, 39(3), 172.
2- Castellao Tavares, S. M. Q. M., Bravo Junior, W. L., & Alves Leite, J. L. (2015). Analyze the levels of immunoglobulins IgG and IgM in elderly and youngs. IntJ Immunol Immunother, 1 (1).
3- Mattos, M. S., Vandendriessche, S., Waisman, A., & Marques, P. E. (2024). The immunology of B-1 cells: from development to aging. Immunity & Aging, 21(1), 54.
Geri bildirim gönder
Yan paneller
Geçmiş
Kaydedilenler

6. PLOS authors have the option to publish the peer review history of their article ([what does this mean?](https://track.editorialmanager.com/CL0/https:%2F%2Fjournals.plos.org%2Fplosone%2Fs%2Feditorial-and-peer-review-process%23loc-peer-review-history/1/010f0192d42d1361-5e782444-d358-4fe0-bf90-a38fd32ff33a-000000/8QRfgcYWc27EqqrZawwArFs3sjdN_UmlyDDd0DjACdQ=182)). If published, this will include your full peer review and any attached files.


If you choose “no”, your identity will remain anonymous but your review may still be made public.


**Do you want your identity to be public for this peer review?** For information about this choice, including consent withdrawal, please see our [Privacy Policy](https://track.editorialmanager.com/CL0/https:%2F%2Fwww.plos.org%2Fprivacy-policy/1/010f0192d42d1361-5e782444-d358-4fe0-bf90-a38fd32ff33a-000000/uGaSqcpzfmRAJJta1wrgd9N3TxT_YP8toPYpoP9334Y=182).

Reviewer #1: No

Reviewer #2: **Yes:** Bekir Kocazeybek
